# Mild Behavioral Impairment association with the Visual Cognitive Assessment Test (VCAT) in a Southeast Asian Population with Vascular Mild Cognitive Impairment ‐ Biomarkers and Cognition Study, Singapore BIOCIS

**DOI:** 10.1002/alz.092216

**Published:** 2025-01-03

**Authors:** Yi Jin Leow, Pricilia Tanoto, Ashwati Vipin, Mohammed Adnan Azam, Gurveen Kaur Sandhu, Faith Phemie Hui En Lee, Smriti Ghildiyal, Shan Yao Liew, Isabelle Yu Zhen Tan, Nagaendran Kandiah

**Affiliations:** ^1^ Lee Kong Chian School of Medicine, Nanyang Technological University, Singapore Singapore

## Abstract

**Background:**

The Visual Cognitive Assessment Test (VCAT) is a visual based, language neutral cognitive assessment validated across cultures and ethnicities, that allows for early diagnosis of cognitive impairment. The VCAT assess domains of cognition – Memory, Visuospatial function, attention, language and Executive function. The Mild Behavioural Impairment Checklist (MBI‐C) was developed to assess five domains of NPS – Decreased motivation, emotional dysregulation, impulse control, social inappropriateness and abnormal beliefs/perceptions Current research indicates an association between MBI symptom severity and poorer cognitive performance in the domains of memory. We examined the association between mild behavioural impairment and domains of cognition as assessed by the VCAT in a multi‐ethnic Southeast Asian population.

**Method:**

204 participants with vascular mild cognitive impairment (VMCI) were recruited from the community living in Singapore. VMIC was defined by Fazekas White Matter Hyperintensity(WMH) visual ratings of 4 and above. The mean age = 66.82 years, mean education = 13.54years with 39.8% males. Participants underwent neuropsychological assessments including the VCAT administered by trained raters and self‐reported questionnaires including the Mild Behavioural Impairment‐Checklist (MBI‐C).

**Result:**

The VCAT‐Attention domain had a significant negative correlation with MBIC‐social subdomain (r = ‐0.276, p<0.05). With every 1 point increase in VCAT‐Attention. MBIC‐social subdomain decreased by 9% (estimate ‐0.357; 95% CI[0.516, 0.239]; p = 0.022).

**Conclusion:**

Current research indicated that social isolation is one of the biggest risk factors for cognitive impairment, and our results reveal that social issues are associated with behavioural impairment as well. Attentional difficulties could lead to issues in inhibitory control, resulting in social inappropriateness. The V‐CAT’s ability to associate specific subdomains with MBI‐C subdomains suggest both tools could be a valuable tool for early detection and intervention in cognitive disorders.

